# Long-Term Effects of Maternal Smoking on Quality of Life. Results from the Copenhagen Perinatal Birth Cohort 1959–61

**DOI:** 10.1100/tsw.2003.59

**Published:** 2003-08-18

**Authors:** SØren Ventegodt, Joav Merrick

**Affiliations:** The Quality of Life Research Center, Teglgårdstræde 4-8, DK-1452 Copenhagen K, Denmark; National Institute of Child Health and Human Development, Ministry of Social Affairs, Jerusalem, Israel

**Keywords:** birth cohort, longitudinal study, maternal health, child health, development, quality of life, maternal smoking, Denmark

## Abstract

The Copenhagen Perinatal Birth Cohort 1959–61 is a prospective longitudinal perinatal study that included all deliveries (over 20 weeks gestation, birthweight over 250 g) that took place at the University Hospital (Rigshospitalet) in Copenhagen, Denmark during the period of September 21, 1959 to December 21, 1961 and used in this follow-up study to investigate the connection between maternal smoking during pregnancy and the quality of life of the child 31 to 33 years later. The latest follow-up study from the cohort was performed in 1993 and 7,222 of the surviving children were identified (now aged between 31 and 33 years) and contacted with a nonanonymous questionnaire on several aspects of quality of life issues.There were 4,626 usable responses (f = 2,489, m = 2,131) corresponding to a response rate of 64.1%. The children whose mothers were nonsmokers or smoked less than three cigarettes a day had a quality of life that was 2.7% better than those children whose mothers had smoked over ten cigarettes per day. At first glance these figures seem small; however, when compared with other early life factors we see that mothers smoking more than ten cigarettes per day is one of the most important early predictors in our study for the quality of life (QOL) of the child as an adult. As most people in our study have a QOL rating between 55% and 85%, 2.7% is about 10% of normal variation. It seems that exposure to tobacco smoke during pregnancy has a small but significant effect on the quality of life in later adult life. However, the underlying causal factor for this reduction in quality of life remains unclear. Nevertheless, pregnant mothers should be made aware of the potential long-term effects smoking can have on their children.

## INTRODUCTION

The adverse effects of smoking on the fetus and the newborn are well known, such as low birth weight, increased risk of spontaneous abortion, increased rate of neonatal death and spontaneous infant death[1]. Furthermore, there is growing evidence to suggest that exposure to tobacco is linked to inhibited physical and mental development and may extend to an increased chance of the child having behavioural problems in the early school years[2,3].

However, less is known about the long-term effects of smoking on the fetus[3]. Does tobacco exposure in early life effect matters of subjective well-being and satisfaction in later life? Or, is the affected child, by the time of adulthood, able to overcome this early influence?

In this presentation we used a large birth cohort study sample in order to investigate the long-term effects on quality of life (QOL) when the mother had smoked during pregnancy.

## METHODS

### Population

The Copenhagen Perinatal Birth Cohort 1959–61[4,5,6,7] is a prospective longitudinal perinatal study that included all deliveries (over 20 weeks gestation, birthweight over 250 g) that took place at the University Hospital (Rigshospitalet) in Copenhagen, Denmark during the period of September 21, 1959 to December 21, 1961. The cohort consisted of 9,006 pregnant women who delivered 8,820 live born infants and 362 stillborn or late abortions over 250 g. 170 women gave birth to twins, three to triplets and the remainder were single births. The pregnant women were contacted and examined before delivery as early in pregnancy as possible. In order to evaluate and code the social, medical, and obstetrical information uniformly, all examinations were done by the same physician[4]. He also met with all women after delivery and coded information recorded by the women themseles during pregnancy and also the information describing the events in the delivery room[7].

The infants were examined by one of three pediatricians on the first and fifth days. Physical and neurological examinations were conducted. Upon discharge the mothers received a questionnaire related to child development during the first year, which they brought to the hospital at the age of 1 year for a physical reexamination (a total of 8,425 children survived 28 days)[5,6,7]. Follow-up physical examinations took place at 3 and 6 years with developmental recording done by the mothers between examinations. At a later stage all school health records were retrieved and coded by one physician[8]. More than 1,000 factors relating to pregnancy, birth, and child development were collected on each child resulting in numerous scientific publications over the last 40 years.

In 1993 a new follow-up study was performed and 7,222 of the surviving children were identified (now aged between 31 and 33 years) and contacted with a nonanonymous questionnaire. A written reminder to nonresponders was sent a month later resulting in 4,626 usable responses (f = 2,489, m = 2,131) corresponding to a response rate of 64.1%. The response rate for each individual question was typically a little lower.

## The Questionnaire

The questionnaire used in the study, "Questionnaire for the Self Evaluation of Quality of Life" (SEQOL), contained 317 questions with 205 in an easy-to-answer multiple-choice format. The questionnaire was divided into the following sections: social information, lifestyle, illness, sexuality, self-perception, life perception, and eight series of questions measuring the quality of life[9].

The development of the questionnaire and progression of the study was guided by the following methodological demands for quantitative questionnaire-based quality of life research[9]:

1. A clear definition of the quality of life.
2. A philosophy of life based on the definition of point 1.
3. A theory that has this philosophy as its framework by
    a) deducing questions that are unambiguous, mutually exclusive, and together are fully exhaustive, and by
    b) establishing the relative weighting of each question.
4. A number of response options that might be given a quantitative interpretation on a fraction scale.
5. Technical checks in terms of reproducibility, sensitivity, well-scaledness, etc.
6. The survey must be meaningful to both researchers, respondents, and those who use the results.
7. An appreciation of the aesthetic dimension.

The present study followed these requirements[9] with the theoretical basis for quality of life measurement based upon the integrative quality of life theory[9]. It organises eight individual theories of quality of life into a spectrum ranging from subjective (self-evaluated) to objective (externally evaluated) quality of life and spanning a core of theories that consider quality of life as deriving from human nature or human existence itself (existential theories). These eight theories or dimensions of life quality were operationalized into eight rating scales, then grouped into three dimensions:

**I. Subjective Dimensions**
1. Immediate, self-experienced well-being
2. Satisfaction with life
3. Happiness

**II. Existential Dimensions**
4. Needs fulfillment
5. Subjective experience of objective temporal domains (family, work, leisure)
6. Subjective experience of objective spatial domains (satisfaction with social relationships)
7. Expression of life's potentials

**III. Objective Dimension**
8. Objective factors (income, employment, education, etc.)

Eighty-five of the questions in the questionnaire were used to measure quality of life along these eight dimensions. A Likert-scale with five response options was symmetrically arranged around a neutral midpoint. As an example, well-being was measured by the question, "How are you feeling now?", and the response options given were "very good," "good," "neither good nor poor," "poor," and "very poor." The central and precisely worded mid-point (neither good nor poor), the response options symmetrically aligned up and down the scale (good, poor) and the use of the same amplifier (very) all combine to suggest that the five points on the scale may be considered equidistant.

If an underlying scale was selected ranging from 0 to 100%, from the worst imaginable to the best imaginable quality of life, the five response options may be reasonably positioned at 10, 30, 50, 70, and 90%. In other words, if a respondent checks "good," his or her well-being is measured as 70%. In this manner, an approximated ratio scale was obtained, so that means could be computed and compared.

A weighted mean for the eight quality of life dimensions was computed by subjective and existential measures, respectively. The resulting overall measure was global (covers all aspects of life, not merely health-related aspects) and generic (not disease-related or intended for a specific category of patients).

Significance levels for the relationships between each variable and the measured quality of life were computed for the continuous variable using classical correlation and a modified regression[9], while in the case of the discrete variables, every group was tested individually against the rest of the sample HO: $\mu i = \mu non\text{-}i$ (that is, the null hypothesis that the mean quality of life for a particular group [for example, smokers] was significantly different from the mean quality of life for the rest of the population [for example, those that do not smoke]).

The questionnaire has been validated[9,10,11,12], and the measurement instruments (the rating scales) proved to be valid and sensitive to the same degree as commonly recognised international instruments. A 1-month and a 3-month test-retest for reproducibility showed correlation coefficients for the eight instruments ranging from 0.6 to 0.9. A qualitative assessment of the validity of the questionnaire was performed, in which 80% of the respondents indicated that the questionnaire items expressed all dimensions relating to their quality of life, 17% were in doubt and 3% felt they did not express all dimensions, which was found to be acceptable.

## RESULTS

In this study quality of life was examined across the eight dimensions discussed above, ranging from the subjective to the objective, while information on the smoking habits of the mother was taken from self-reported data, collected during the paediatric study.

In Table 1 the percentage deviation from the mean quality of life is expressed as a percentage of that mean and we found a small but significant connection between the smoking of the mother in the last trimester of pregnancy and the quality of life of the child 31 to 33 years after birth. The children whose mothers were nonsmokers or smoked less than three cigarettes a day had a quality of life that was 2.7% better than those children whose mothers had smoked over ten cigarettes per day.

For a clearer understanding of these results it may have been appropriate to continue the analysis with standard procedures such as partial regression controlling for social factors, mother's health or the person's own tobacco consumption, but these procedures are themselves a source of "noise," so the value of such tests is limited.

**TABLE 1**
**Percentage Deviation from the Mean Quality of Life Expressed as a Percentage of That Mean**

| | % | 1 | 2 | 3 | 4 | 5 | 6 | 7 | 8 | Overall deviator from QL Mean (weighted*) | Test (p-value) |
|---|---|---|---|---|---|---|---|---|---|---|---|
| Less than 3 cigarettes a day | 56.4 | 0.0 | 0.0 | 0.3 | 0.4 | –0.2 | 0.4 | 0.2 | 1.2 | 0.5 | * 0.0169 |
| 3 to 10 cigarettes a day | 29.7 | 0.3 | 0.6 | 0.0 | 0.2 | 0.3 | 0.1 | 0.8 | –0.4 | 0.1 | 0.8936 |
| More than 10 cigarettes a day | 13.8 | –1.1 | –2.1 | –1.7 | –2.5 | –0.7 | –2.1 | –2.4 | –2.9 | –2.2 | ** 0.0011 |

Columns: % - the percentage of the 4626 respondents in the group; "1" – Immediate, self-assessed well-being; "2" – Life satisfaction; "3" – Happiness; "4" – Fulfilment of needs; "5" – Experience of objective, temporal domains (family, work, leisure); "6" – Experience of objective, spatial domains (self, other, world); "7" – Expression of life's potentials; "8" - Objective factors ; the overall deviation from average in the total QOL score; the resulting p value. The numbers in the table are the deviations from the average QOL scores in percent. The total QOL is calculated according to the IQOL theory [see 9] as (("1"+"2"+"3"):3 + ("4"+"5"+"6"+"7"):4 +"8"):3.

## DISCUSSION

From the investigation, in which over 1,000 early-life factors were examined, maternal smoking was one of the 50 factors that actually had a significant, detrimental effect on the quality of life of the child 31 to 33 years later.

At first glance these figures seem small. However, when compared with other early life factors, we see that mothers smoking more than ten cigarettes a day is one of the more important early predictors in our study, for the QOL of the child as adult. As most people in our study have a QOL rating between 55 and 85%, 2.7% is about 9% of normal variation. Because there seems to be a strong connection between QOL and general performance in life as QOL is a measure for immediately accessible personal resources, the 2.7% in global QOL might indicate an important reduction in performance both in private and professional life, for the children of heavy smokers during pregnancy, although this also should be an issue for further research.

What we are unable to see from the data is whether the reduction in quality of life is caused by the actual effects of the tobacco smoke or whether maternal smoking is just an indicator of other causal factors. In the prospective longitudinal cohort study of 12,068 pregnant women and their children from Northern Finland in 1966 and 9,362 mothers from another birth cohort started in 1985–86, 12% and 18%, respectively, of the women in the two cohorts continued smoking during the pregnancy[13]. The follow-up at 14 and 21 years showed a more favourable child development for the group of mothers that were nonsmoking or stopped smoking during pregnancy. Follow-up at age 28 of the 1966 Finnish birth cohort study[14] was concerned with the relationship between maternal smoking and the risk for criminal behaviour, and found that for the sons of mothers who had smoked during pregnancy had a twofold risk of having committed a violent crime or having repeatedly committed crimes, even when other biopsychosocial risk factors were controlled. When maternal smoking during pregnancy was combined with a maternal age below 20 years, single parent status, unwanted pregnancy, and developmental lag in walking and talking, the odds ratio for violent offenses increased up to 9-fold and for persistent offenses up to 14-fold[14]. Some of these findings are the sort of objective factors that could account for the loss of quality of life we are witnessing.

However, there is also considerable evidence to suggest that chemicals in tobacco smoke are capable of producing detrimental changes in the placenta and fetus[15] and these changes could be linked to small cognitive and achievement deficits and perhaps even mental retardation[16], which again could be an explanation for the reduction in quality of life.

If maternal smoking was just an indicator for social status in Denmark around the 1960s, mothers' smoking in itself might not harm the child at all. On the other hand, a worst case scenario is that children of heavy smoking mothers might be not only even less happy, but also about 9% poorer performers than children of nonsmoking mothers; this could be enough to give them an important handicap in a competitive society.

As maternal smoking is known to correlate with the size of the child's brain, a fair hypothesis for further research is that the observed effect is caused by the effect of the highly toxic carbon monooxide (CO) from the cigarettes on the developing brain of the fetus. The idea that cigarettes disturb the development of the fetal brain to such an extent that the child as an adult will have a severe competitive handicap in society is fairly frightening.

## CONCLUSION

It seems that fetal exposure to tobacco smoke has a small albeit significant and presumably important effect on the quality of life in adulthood. However, the underlying causal factor for this reduction in quality of life remains unclear. Nevertheless, there still exists every reason to make pregnant mothers aware of the potential long-term effects smoking can have on their children.

Unfortunately there is a scarcity of data on maternal smoking, due to the paucity of perinatal cohorts aged 30 years. We sincerely hope that the world's mothers, in spite of the sad incompleteness of the scientific picture, will take seriously the data we provide in this article and stop smoking during pregnancy.

## ACKNOWLEDGEMENTS

This paper reported results from the Danish Quality of Life Population Survey, the planning and execution of which would not have been possible without the aid of Dorte Loldrup Poulsen, Jørgen Hilden and the late Bengt Zachau-Christiansen. Sources of support came from the The 1991 Pharmacy, the Goodwill, the JL, E. Danielsens & Wife's, Emmerick Meyer's, the Frimodt-Heineken, the Family Hede Nielsen's, Petrus Andersen's, C.P. Frederiksen's and the Wedell-Wedellsborgs Foundations, and IMK Almene Fond. The research was approved by the Copenhagen Ethical Committee under numbers (KF) V 100.1762/90 and (KF) 01-502/93.

**BIOSKETCH**

**Søren Ventegodt, MD,** is the director of the the Quality of Life Research Center in Copenhagen, Denmark. He is also responsible for a research clinic for holistic medicine in Copenhagen and used as a popular speaker throughout Scandinavia. He has published numerous scientific and popular articles and a number of books on holistic medicine, quality of life, and quality of working life. His most important scientific contributions are the comprehensive SEQOL questionnaire, the very short QoL5 questionnaire, the integrated QOL theory, the holistic process theory, the life mission theory, and the Danish Quality of Life Research Survey, 1991–94 in cooperation with the University Hospital of Copenhagen and the late pediatric professor Bengt Zachau-Christiansen. Website: www.livskvalitet.org/

**Joav Merrick, MD, DMSc,** is professor of child health and human development affiliated with the Zusman Child Development Center and Division of Community Health at the Ben Gurion University, Beer-Sheva, Israel and presently the medical director of the Division for Mental Retardation, Ministry of Social Affairs, Jerusalem and the director of the National Institute of Child Health and Human Development. He has authored and edited numerous publications in the fields of child and human development, rehabilitation, intellectual disability, disability, health, welfare, abuse, advocacy and prevention. Dr. Merrick received the Peter Sabroe Child Award for outstanding work on behalf of Danish Children in 1985 and the International LEGO-Prize ("The Children's Nobel Prize") for an extraordinary contribution toward improvement in child welfare and well-being in 1987. Website: www.nichd–israel.com
